# The Complex Tumor Microenvironment in Ovarian Cancer: Therapeutic Challenges and Opportunities

**DOI:** 10.3390/curroncol31070283

**Published:** 2024-07-01

**Authors:** Bianca Garlisi, Sylvia Lauks, Caroline Aitken, Leslie M. Ogilvie, Cielle Lockington, Duncan Petrik, Jan Soeren Eichhorn, Jim Petrik

**Affiliations:** Department of Biomedical Sciences, University of Guelph, Guelph, ON N1G 2W1, Canada; bgarlisi@uoguelph.ca (B.G.); slauks@uoguelph.ca (S.L.); caitke01@uoguelph.ca (C.A.); ogilviel@uoguelph.ca (L.M.O.); clocking@uoguelph.ca (C.L.); petrikd@uoguelph.ca (D.P.); jeichhor@uoguelph.ca (J.S.E.)

**Keywords:** tumor microenvironment, ovarian cancer, immunosuppression, interstitial fluid pressure, vascular normalization, tumor angiogenesis

## Abstract

The tumor microenvironment (TME) in ovarian cancer (OC) has much greater complexity than previously understood. In response to aggressive pro-angiogenic stimulus, blood vessels form rapidly and are dysfunctional, resulting in poor perfusion, tissue hypoxia, and leakiness, which leads to increased interstitial fluid pressure (IFP). Decreased perfusion and high IFP significantly inhibit the uptake of therapies into the tumor. Within the TME, there are numerous inhibitor cells, such as myeloid-derived suppressor cells (MDSCs), tumor association macrophages (TAMs), regulatory T cells (Tregs), and cancer-associated fibroblasts (CAFs) that secrete high numbers of immunosuppressive cytokines. This immunosuppressive environment is thought to contribute to the lack of success of immunotherapies such as immune checkpoint inhibitor (ICI) treatment. This review discusses the components of the TME in OC, how these characteristics impede therapeutic efficacy, and some strategies to alleviate this inhibition.

## 1. Introduction

### 1.1. OC Biology and Subtypes

OC is the most lethal gynecologic malignancy and the fifth leading cause of cancer-related death in women. Due to its vague, non-specific symptoms and the lack of effective screening strategies, OC is often not diagnosed until advanced stages of disease, where treatments have reduced efficacy, and the 5-year survival rate is poor. Therapeutic strategies for advanced OC have remained unchanged for decades and typically involve cytoreductive surgery followed by platinum- and taxane-based chemotherapy. The inhospitable and immunosuppressive ovarian TME presents several potent barriers to chemotherapeutic success, frequently resulting in therapy resistance and disease relapse. Thus, there is a need for innovative approaches to improve the management and prognosis of this devastating disease. This review focuses on a description of the OC microenvironment, its impact on therapeutic success, and potential avenues to address these issues.

### 1.2. Classification and Histopathology

**Figure 1:**
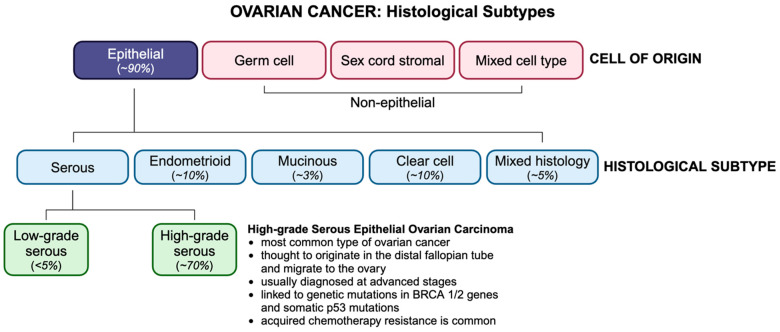
OC histotypes and their incidence. OC is classified based on their cell type of origin, with HGSC being the most common subtype.

OC is classified by histological subtype, according to which ~90% of OCs are of epithelial origin (Figure 1). Non-epithelial subtypes include germ cell and sex cord stromal cancers, which comprise less than 10% of all OCs and are associated with a more favourable prognosis. Epithelial ovarian tumors have three origin sites, namely ovarian, tubal, or other epithelial sites within the pelvis. Epithelial ovarian malignancies are divided into type I and type II tumors and include four primary histologic subtypes, namely serous, endometrioid, mucinous, and clear cell. Serous carcinomas are categorized as high-grade (HGSCs) or low-grade serous carcinomas (LGSCs), of which HGSCs account for ~75% of all epithelial subtypes and are known as the most aggressive and fatal ovarian malignancies. Unlike type I tumors, which are thought to originate in the ovaries, type II tumors tend to originate in the distal fallopian tube epithelium and migrate to the ovary. Type II tumors, which are frequently linked to germline mutations of the BRCA genes or somatic p53 mutations, are usually diagnosed at stage III, when cancer has spread beyond the pelvic cavity, and the 5-year survival rate is less than 30% [1].

### 1.3. Diagnosis and Standard of Care

Due to the presence of vague, non-specific symptoms (e.g., abdominal bloating/pain, feeling full, and urinary frequency), many women are not prompted to seek medical care, resulting in diagnosis at an advanced stage. Screening for OC is challenged by the lack of markers with sufficient sensitivity and specificity. As such, early detection for ovarian malignancies remains an unmet medical challenge. OC diagnosis typically involves transvaginal sonography, which is useful in screening for pelvic masses, and a blood test for cancer antigen 125 (CA125); however, conclusive diagnosis of malignancy requires a tissue biopsy.

Standard first-line treatment for advanced HGSC OC involves cytoreductive surgery with dose-dense carboplatin and paclitaxel chemotherapy. Despite initial responsiveness to chemotherapy in ~70% of women, within 3 years of primary therapy, the majority of patients develop disease recurrence and become refractory to chemotherapy [2]. This therapeutic approach has not changed appreciably in decades and highlights a desperate need for novel treatment strategies to improve the prognosis for women diagnosed with OC. 

Despite ongoing clinical trials and continuous scientific discovery, OC persists as one of the most lethal malignancies in women [3]. Unfavourable clinical outcomes are primarily attributed to the complex and heterogeneous nature of the TME [4]. Many cancer treatments aim to target cancer cells while ignoring surrounding tumor components, and the heterogeneous TME can potentiate therapy resistance and subsequent tumor recurrence [4]. Thus, the intricate characteristics of the OC TME pose challenges for therapeutic intervention. 

## 2. The TME

The TME is composed of cellular and noncellular constituents, including fibroblasts, endothelial cells, and immune cells, as well as the extracellular matrix (ECM); ECM remodeling enzymes, such as matrix metalloproteinases (MMPs); growth factors, including vascular endothelial growth factor (VEGF), transforming growth factor beta (TGF-B), and platelet-derived growth factor (PDGF); and the tumor stroma, respectively [5]. The stroma has a profound impact on many hallmarks of cancer and plays a crucial role in tumorigenesis, therapy resistance, and metastasis [4]. The ECM is composed of collagens, glycoproteins, and proteoglycans, which provide structural support but also regulate cellular function within ovarian tumors [6]. Proteins and secreted factors associated with the ECM are important in providing biochemical and biomechanical stimuli that regulate the function of many cell types within the tumor [7,8]. The ECM impacts cell function by activating receptors such as integrins and syndecans and can have indirect effects by modulating receptor binding site availability or proximity [9]. Tumor cells have abnormal ECM deposition, which can alter physical characteristics of the cells, often increasing mechanical stiffness, inducing proliferation, invasion, and resistance to cell death [10]. Due to the reciprocal interaction between cancer cells and the ECM, targeting the ECM as a therapeutic approach has shown some preclinical promise but no significant clinical impact to date [11]. The presence of a significant stromal component in OC has been associated with poor prognosis of advanced-stage OC [12]. Tumor cells orchestrate the recruitment and activation of stromal cells within the TME and release inflammatory and pro-fibrotic factors, including TGF-B, PDGF, and fibroblast growth factor-2 (FGF-2), amongst others, inducing differentiation of stromal cells into CAFs [13,14]. CAFs are essential in the development of desmoplasia and are involved in the remodeling of the TME to facilitate cancer progression [15]. Fibroblasts become activated under stressful conditions and are recruited in response to epithelial damage, after which they begin secreting signal mediators to guide wound healing and immune functions [16]. Normal activated fibroblasts are supposed to be cleared through apoptosis after the tissue is healed [17], but some cells resist this apoptotic signal and are labeled CAFs [16]. CAFs are a heterogenous population that promotes tumor development through different mechanisms [18]. More specifically, CAFs release MMPs that degrade components of the ECM, including fibronectin and collagen [15]. In OC, the MMP family has been shown to play a role in promoting tumor growth and invasion, inflammation, and angiogenesis [19]. MMP-2 and MMP-9 protein expression in stromal cells was found to be significantly related to advanced-stage disease and poor prognosis [20]. The abundance of CAFs, pericytes, and mesenchymal stem cells (MSCs) in the TME has also been associated with late-stage disease, resistance to treatment, and increased lymphatic and microvessel density [21].

## 3. Tumor Vasculature

Angiogenesis is the process by which new blood vessels form from pre-existing vasculature and is crucial for the continued growth of tumors and survival of cancer cells. Without initiating angiogenesis, tumors cannot exceed 1–2 mm^2^ due to limited nutrient and oxygen supplies and a lack of waste removal [22]. In the absence of adequate vascularization, tumor cells become necrotic [22]. In a quiescent tumor, pro-angiogenic factors such as VEGF-A are counter-balanced by anti-angiogenic factors such as angiostatin and thrombospondin [23]. For tumor angiogenesis to occur, there needs to be an “angiogenic switch” in which there is an upregulation of pro-angiogenic factors and a concomitant suppression of anti-angiogenic factors within the tumor. Notably, in response to hypoxic conditions, hypoxia-inducible factors (HIFs) become active [23]. In response to tumor hypoxia, HIF-1α is stabilized, resulting in the upregulation of pro-angiogenic factors, including VEGF, PDGF, and FGF-2 [23]. Cathepsin proteases have been implicated in the remodeling of the tumor ECM and the promotion a permissive environment for vessel development and migration [24,25]. Cathepsin L promotes metastatic migration of EOC cells to the omentum by increasing omental angiogenesis [26], at least partly by inducing the expression of galectin-1 in human omental microvascular endothelial cells, driving proliferation and increased vessel density [27]. Cathepsin D is pro-angiogenic as well, inducing blood vessel formation in a chick chorioallantoic membrane CAM assay [28], and has been shown to cleave pro-angiogenic factors from the ECM in breast cancer [29]. Part of the mechanism by which Cathepsin L and D initiate tumor angiogenesis is thought to be through regulation of pericyte recruitment and function within the TME [30]. The increased production of pro-angiogenic factors facilitates the “angiogenic switch”, which transitions the tumor to a pro-angiogenic and pro-tumorigenic state [23]. CAFs also contribute to tumor angiogenesis through the production of MMPs, which stimulate the degradation of the vascular basement membrane, leading to endothelial cell proliferation and migration [15]. As MMPs degrade the ECM, they result in the release of sequestered angiogenic factors such as VEGF from the ECM, increasing bioavailability and stimulating new capillary formation within the tumor [31]. The simultaneous HIF-1-induced VEGF upregulation and increased levels of MMP-9 result in high concentrations of soluble VEGF, driving angiogenesis and metastasis [32]. While the pro-angiogenic stimulus is designed to increase tumor microvessel density, this pro-angiogenic stimulus is so aggressive that the result is the rapid and chaotic formation of blood vessels, resulting in malformed and heavily fenestrated, leaky vasculature. These poorly formed vessels paradoxically result in poor tumor perfusion and areas of tumor hypoxia, while the excessive vessel fenestration facilitates tumor cell intravasation and metastatic spread through the vascular system [33,34]. Increased intra-endothelial cell spaces result in elevated permeability and increased IFP [35]. Reduced perfusion and elevated tumor IFP create significant barriers to therapy uptake. Abnormal angiogenesis in tumors also creates an immunosuppressive environment, limiting tumor-infiltrating lymphocyte (TIL) infiltration and promoting the prevalence of pro-tumor lymphocytes [36].

### Tumor Metabolism

OC cells have accelerated rates of proliferation and are very metabolically active, which can lead to an acidic TME [37]. Cancer cells, even in the presence of oxygen, preferentially metabolize glucose through anaerobic glycolysis in a process known as the Warburg effect [38]. This switch from aerobic to anaerobic metabolism gives cancer cells a survival advantage in a hypoxic environment, protecting against hypoxia-induced apoptosis and oxidative damage [38]. In addition to aerobic glycolysis facilitating tumorigenesis, drug resistance, and metastasis, the quantities of the produced lactic acid also acidify the TME [39]. An acidic TME has been shown to contribute to aggressive tumorigenesis, invasiveness, and therapy resistance in many types of cancer [39,40].

## 4. Stromal Compartment

The stroma has important roles in modifying the environment of the ovaries to support malignant cells and create a permissive TME. The stroma consists of non-malignant cells and connective tissue surrounding cancerous cells. The cells of the stroma include CAFs, mesenchymal cells, immune cells, endothelial cells, and pericytes, as well as the ECM [41]. An important cell type within the stroma is recruited macrophages, which are the main population of immune cells within the stroma [42]. These macrophages differentiate into two different classes, namely the M1 phenotype, which is the less tumorigenic form, and the M2 phenotype, which is associated with tumor progression [43]. These M2 macrophages are referred to as TAMs when they show pro-tumor functions such as increased cell proliferation, survival, and metastasis [43]. TAMs pose a direct challenge to therapy through the secretion of chemoprotective molecules such as cathepsin b, in addition to enhancing the angiogenic capability of the tumor [44]. TAMs communicate with other non-cancerous cells to coordinate the formation of the TME, and fibroblasts are a prominent cell type involved in this crosstalk. Fibroblasts are spindle-shaped cells found in connective tissues and are broadly categorized by their response to tissue injury and contributions to tissue homeostasis [45]. As part of the response to injury, fibroblasts secrete many different chemical signals, such as chemokines for pericyte and endothelial cell recruitment during angiogenesis [41]. For normal tissue homeostasis, fibroblasts produce and deposit various types of collagen and other connective tissues in the ECM, as well as matrix proteins such as MMPs [41]. Fibroblasts express certain growth factors, such as epidermal growth factor and hepatocyte growth factor (HGF), which are common initiators of cell proliferation of epithelial cancer cells [46]. The function of HGF is further corrupted by the tumor to regulate metabolism, creating an acidic environment [47]. Within the cortical region of the ovaries, there is an abundance of connective tissues, myofibroblasts (an activated phenotype of fibroblast), and fibroblasts [48]. Once a tumor develops, the local ovarian fibroblasts become the main source of CAFs in the primary tumor [49]. Interestingly, local ovarian fibroblasts are not found within the secondary metastasis, which suggests a different source for CAFs in secondary tumors [49]. The most prevalent population of CAFs is myCAFs, which include traditional myofibroblasts and have specific roles in the contraction and stiffening of tissues through the expression of alpha smooth muscle actin [50]. Another important phenotype is iCAFs, which are inflammatory CAFs that express key mediators of inflammation such as Ly6C [51], similar to inflammatory macrophages [50]. CAFs also secrete CC chemokine ligand 2 (CCL2), which binds to the CCR2 receptor on macrophages, recruiting macrophages into the stromal compartment [52]. Stromal-derived factor-1 (SDF-1) secreted by CAFs promotes the M2 differentiation of macrophages, resulting in the formation of TAMs [52]. This initiates a positive feedback loop where TAMs enhance the mesenchymal-to-epithelial transition (MET) of fibroblasts and stimulate their activation into reactive CAFs [53]. Another challenge posed by CAFs is their contributions to immune evasion and the generation of an immunosuppressive environment within the stroma of solid tumors. The immunosuppressive environment is enhanced by CAFs through recruitment, activation, enhanced survival, and differentiation of CD4+CD25+FOXP3+Tregs, which inhibit an immune response [54]. Similarly, many other myeloid cells are recruited, and their functional differentiation into tumor-promoting/immunosuppressive phenotypes is regulated by CAF action [54]. Mechanically, CAFs modify the stroma through aberrant ECM remodeling to promote tumor progression and inhibit immune cell infiltration. SDF-1, secreted by CAFs, increases MMP production, which facilitates tumor invasion by degrading surrounding tissues [47].

## 5. Immune Environment in Ovarian Tumors

### Immune Cells in the TME

**Figure 2:**
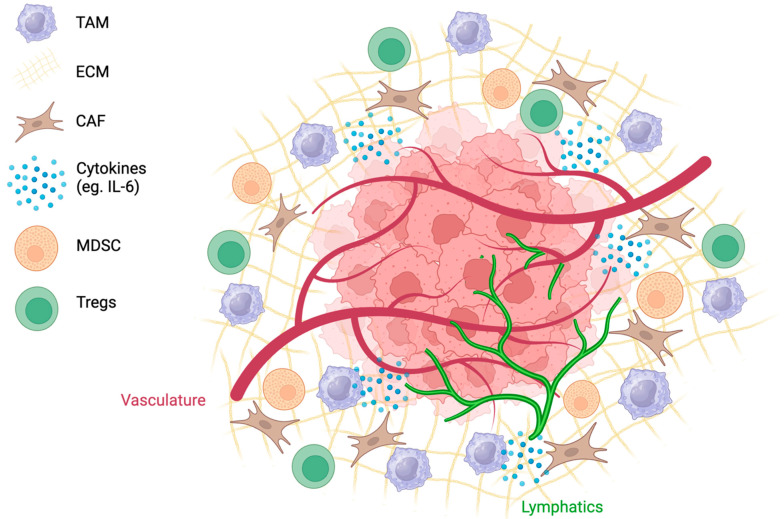
The TME is complex, with tumor and lymphatic vessels, a dense ECM, and highly expressed cytokines. Recruitment of cells such as CAFs, TAMs, MDSCs, and Tregs creates an immunosuppressive environment.

The complex interplay of abnormal angiogenesis, hypoxia, and acidity within the TME is associated with the recruitment of immunosuppressive cell populations (Figure 2). Tumor cells release danger-associated molecular pattern molecules that enable the recruitment of immune cells into the TME [55]. Innate immune cells, such as natural killer cells, macrophages, and dendritic cells, as well as tumor-infiltrating lymphocytes (TILs), such as CD8+ and CD4+ cells and B lymphocytes, are recruited to eliminate cancer cells [55]. Typically, CD3+, CD4+, and CD8+ TILs are associated with a favourable clinical outcome [56]. In a study analyzing 186 tumors from advanced-stage OC patients, patients with CD3+ TILs had a five-year survival rate of 38%, in contrast to 4.5% for patients without detectable TILs [56]. Cancer cells can avoid immune destruction through immunoediting and survive in equilibrium with effector T cells while in a pro-inflammatory environment [14]. Although the mechanisms behind immunoediting have not been fully elucidated, changes in the immunogenicity of the tumor are accompanied by the loss of Ag expression and MHC molecules [57]. These tumors trigger an immunosuppressive environment through the recruitment of MDSCs, TAMS, and Tregs [14].

Lymphatic vessels are also important in the regulation of tumor immunity. In addition to important interstitial fluid drainage, lymphatic vessels provide an essential conduit for immune cells and other factors to migrate to the tumor-draining lymph node (TDLN) [58]. The TDLN is an essential site for tumor antigen exposure [59] and facilitates mobilization of peripheral immunity by activating the adaptive immune response and educating T cells of tumor antigens [59].

However, due to the delicate structure of lymphatic vessels, the elevated IFP within the tumor often collapses these structures, impairing fluid drainage and migration of activated immune cells to the TDLN [60].

OC tumors are often classified as being ‘immunologically cold’, with low numbers of anti-cancer immune cells and disproportionately high numbers of immune-inhibitory cells. This ‘cold’ environment is partly characterized by limited CD8+ T lymphocyte activation and subsequent infiltration into ovarian tumors [61,62]. Many OC tumors have a low tumor mutational burden (TMB) [63], and as these mutations act as targets for antigen-presenting cells (APC), there can be a muted anti-tumor immune response. With dysregulated tumor vasculature and a decreased number of functional tumor lymphatic vessels, the migration of activated cytotoxic lymphocytes to the tumor and APCs to the TLDN is impaired, further contributing to the overall immunosuppression seen in ovarian tumors [64].

The widespread hypoxia within the ovarian TME, in addition to initiating the release of pro-angiogenic factors, is also associated with the recruitment of Tregs, which generally function to suppress lymphocyte recruitment [65]; decrease the maturation of APCs, which are critical for T-lymphocyte activation [65]; and function to promote TAMs [65,66]. A 2021 study analyzing the gene expression profiles of 748 OC patients found that immature dendritic and Tregs were significantly upregulated in tumors with high degrees of hypoxia [67]. HIF-1 directly upregulates programmed cell death-ligand 1 (PD-L1) expression on T cells by binding to HRE in the PD-L1 promoter [68,69]. Higher levels of PD-L1 are associated with accelerated tumorigenesis and poor prognosis [70,71]. Numerous studies in various types of solid tumors have shown that elevated HIF-1 expression contributes to elevated PD-L1 expression in both tumor cells and APCs [69,72,73].

Hypoxia also triggers the release of chemokines that recruit immunosuppressive cell populations, including TAMs, MDSCs, and Tregs, dampening the immune response [14]. A study looking at hypoxia and immune tolerance found that hypoxia influences the expression of chemokine ligands such as CCL28, leading to the recruitment of Tregs and triggering angiogenesis [74]. Additionally, TAMs produce CCL22-recruiting Tregs, which subsequently induces the expression of B7-H4 on APCs, inhibiting cytolytic activity and T-cell proliferation [75,76]. MDSCs and OC cells also produce metabolic enzyme IDO, which breaks down tryptophan, rendering T cells inactive [14].

Tregs further contribute to immunosuppression by expressing the regulatory transcription factor forkhead box protein P3 (FOXP3) and function to suppress an immune response by inducing apoptosis [77] and inhibiting proliferation [78] of CD8+ cytotoxic lymphocytes. One of the prominent mechanisms by which Tregs control this is through the upregulation of immune checkpoints such as PD-L1 and cytotoxic T-lymphocyte-associated protein 4 (CTLA-4) [78], which causes T-cell exhaustion and tolerance, further limiting an adequate response. In response to endogenous anti-tumor activity, tumor cells overexpress PD-L1(21). PD-L1 binds to PD-1 on activated T cells, inhibiting cytotoxic T cells, which remain deactivated in the TME [79]. Similarly, CTLA-4 is constitutively expressed on Tregs and binds to CD80/86 on APCs, sending an inhibitory signal causing T-cell inactivation [79]. Zeng et al. (2019) found that suppressing Tregs and blocking the partnering of PD-L1 and PD-1 led to a significant increase in CD8+ cytotoxic T lymphocytes, promoted the conversion of Tregs to T-helper cells, and resulted in a rise in the ratio of M1 TAMs to M2 TAMs in a murine OC model [80].

Galectin-1 is a member of a family of glycan-binding proteins and is implicated in many aspects of tumorigenesis, including in regulating cell proliferation, adhesion, angiogenesis, and apoptosis and in regulating the TME immune environment [81,82]. Galectin-1 contributes to the immunosuppressive environment, in part, by increasing the number and activity of Tregs in tumors [83] and by skewing CD4+ T cells to the M2 phenotype [84].

TAMs have also been linked to chemoresistance, tumor metastasis, and poor prognosis [85]. TAMs express elevated levels of inflammatory and inhibitory cytokines such as IL-10 and TGF-β and induce tumor angiogenesis by producing growth factors, including VEGF and PDGF [85]. Phenotypically, TAMs adhere to an M1/M2 paradigm where a pro-inflammatory state characterizes M1 macrophages, while M2 macrophages are classified as pro-tumoral and anti-inflammatory [86]. M2 macrophages are typically induced by TGF-β and IL-4/13, secreting anti-inflammatory cytokines that recruit Tregs, further perpetuating an immunosuppressive environment [86]. Ovarian tumors predominantly exhibit a pro-tumor M2 phenotype [85]. Specifically, a study analyzing OC stem-like cells demonstrated that increased COX-2, CCL2, and PGE-2 augmented a shift toward M2 macrophages in the tumor [87]. Opposingly, M1 macrophages mount an anti-tumor response and are associated with favorable outcomes. As such, a high M1/M2 ratio is associated with the improved survival of OC patients [88]. A retrospective study of stage III-IV OC patients analyzed CD68 and CD163 as M1 and M2 macrophage markers [89]. There was a significant difference in overall survival and progression-free survival in the low-CD163 (M2) group [89]. Additionally, a meta-analysis of 794 OC patients across nine studies demonstrated that a tumor’s high M1/M2 ratio predicted an improved prognosis [90]. Ultimately, ongoing research goals include reducing the prevalence of M2 macrophages and increasing M1 counterparts in the TME.

## 6. Barriers in the TME That Inhibit Therapeutic Success

**Figure 3:**
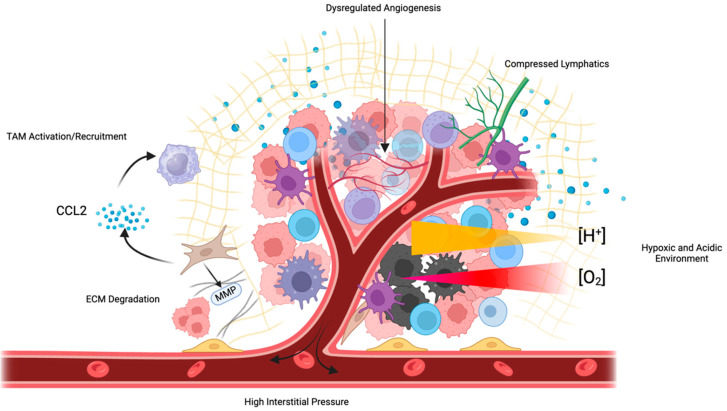
Numerous features of the TME inhibit therapeutic efficacy. Tumors exhibit poorly formed, dysfunctional vasculature, which impedes perfusion and increases IFP, compressing tumor lymphatic vessels. This inhibited perfusion leads to tumor hypoxia and the development of an acidic environment. CAFs and MDSCs secrete immunosuppressive cytokines such as CCL2, which recruit TAMs and impede the efficacy of immunotherapies such as ICIs.

In addition to the use of cytotoxic chemotherapy that persists today, there has been a host of targeted therapies and small-molecule inhibitors developed for the treatment of ovarian cancer (Table 1). The success of these therapies has been variable, often due to resistance mechanisms developed within the tumor. There are several components of the TME that work independently or cooperatively to inhibit the success of a variety of different therapies [91,92] (Figure 3). The TME is involved in the tumor immunosuppressive environment through the activation of a chronic inflammatory state whereby various cytokines, chemokines, and inflammatory mediators suppress the function of both the innate and adaptive immune response [92]. Protective immune cells that are recruited in response to chronic inflammation, such as T helper -2 (Th-2), tend to enhance the function of pro-tumor adaptive pathways such as ICIs PD-1/PD-L1 and CTLA-4 [93]. This inflammatory process acts in a paracrine mechanism to dispatch additional tumorigenic processes such as the recruitment of MSCs, which are multifunctional in impeding many immune cell interactions [94].

The diversity of mechanisms deployed by tumors to evade immune cell anti-tumor pathways suggests the need for combination therapy with treatments that have complementary or synergistic properties [109]. Such combinations include immunotherapy with chemotherapy, the use of anti-angiogenic factors with chemotherapy, and other targeted molecular therapies [110]. Due to the vastness of the array of immunotherapy approaches and the variability of responses between individuals, it has been difficult determine the most effective immunotherapeutic approach for different cancer patients [111]. In OC, the use of ICIs as single agents only has resulted in a response rate between 6 and 15% [112]. Their combined use (anti-PD-1 and anti-CTLA-4) has been reported to result in increased adverse effects and a lack of durable responses in OC patients under both monotherapy and dual immunotherapy. Due to the immunogenic landscape of the TME, the idea surrounding the clinical combination of immunotherapies with cytotoxic chemotherapy has been suggested to provide better survival outcomes [113].

Treatment success can also be determined by specific mutations that give somatic cells fitness advantages in their microenvironment [114]. This is referred to as the TMB, where somatic cell mutations can express antigens that are recognized by the immune system. It has been proposed that solid tumors with a higher TMB are more likely to respond to immunotherapies due to their immunogenicity [115]. Unfortunately, OC has a low-TMB phenotype of roughly 5.3 mutations/Mb, making response prediction and estimates of the likelihood of resistance more challenging [116].

While showing some promise, current therapies have been challenged by the impediments inherent in the TME. However, there are approaches to combat aspects of the TME that inhibit therapeutic efficacy. Reconfiguration of the TME could be an important component of enhancing the efficacy of combination therapies [117].

Immunotherapies have the opportunity to kill tumor cells with enhanced specificity, without the need for systemic cytotoxic drugs. Various immunotherapies, such as ICIs, chimeric antigen receptor T-lymphocyte therapy (CAR-T), vaccine-based therapies, and oncolytic viruses, have already shown promise in clinical studies and are currently approved for use in melanoma, lymphomas, leukemia, lung cancer, and many more diseases, with 12 FDA-approved drugs as of 2023 [118,119]. However, for OC, results have been underwhelming, and there are currently few immunotherapy options for OC patients [117]. However, a number of clinical trials are ongoing, evaluating the impact of novel immunotherapy approaches, either alone or in combination with other therapies [112,116].

ICIs function to block a tumor’s immunosuppressive defense by impairing the activation of immune checkpoints and limiting the opportunity for immunosurveillance [120]. Commonly studied ICIs include PD-L1 and CTLA-4, along with others, including lymphocyte activation gene-3 and T-cell immunoglobulin and mucin-domain-containing-3 [121]. A phase 2 proof-of-concept clinical study looking at the combination of durvalumab (anti-PD-L1) and olaparib (PARP inhibitor) found that the combination offered greater induction of immunostimulatory cytokines [122]. This study also found that platinum-chemotherapy-resistant patients benefitted from combination ICI and PARP inhibition therapy [122]. CAR-T immunotherapy involves the isolation of T cells and the addition of a cancer antigen to increase the “visibility” of cancer cells and achieve a more complete and specific anti-cancer effect. However, selection of the optimal cancer antigen can be difficult and can be highly individualized. In a murine model of OC treated with mesothelin-specific CAR-T cells, Schoutrop et al. (2021) found that these engineered cells were able to significantly increase survival and resulted in better long-term remission, depending on the costimulatory domain used in CAR-T cell development [123]. As with other therapies, CAR-T therapy is hindered by limited access to the tumor [124]. However, vascular normalization can enhance the uptake of CAR-T cells and improve distribution throughout the TME [125]. CAR-T cells are still susceptible to the immunosuppressive TME, as the high numbers of MDSCs and TAMs release immunosuppressive cytokines such as TGFB and IL-10, which suppress their activity [126]. Armored CAR-T cells modified to secrete pro-inflammatory IL-12 [127], Il-15 [128], or IL-21 [129] cytokines have shown enhanced immune activation and greater anti-tumor efficacy. As CAR-Ts are generally hyperactive and difficult to control, there can be a significant cytokine release in response to their infiltration. This rapid cytokine release can result in significant side effects, such as fever, arthralgia, shock, multi-organ failure, and possibly death [130]. In OC, CAR-T therapy has not yet been studied extensively in clinical trials. However, a trial with second-cohort dosing is underway with follicle-stimulating hormone receptor (FSHR T)-mediated T cells (NCT05316129) in patients with recurrent/resistant OC that has progressed under the previous two therapies. Another active trial (NCT04670068) is underway to evaluate the efficacy of CAR-T cells with the B7-H3 antigen (CAR.B7-H3 T cells) in recurrent OC. Although these trials are in early stages, there is hope that efficacy will be achieved in recurrent OC, which is notorious for being extremely difficult to treat.

Another anti-cancer immunotherapy involves the development of cancer vaccines. There is a variety of cancer vaccine strategies, including dendritic cell (DC) vaccines, in which a patient’s DCs are primed with a cancer antigen so that when the DCs migrate to the TDLN, the antigen is presented to T cells, resulting in the activation of peripheral immunity against cancer cells expressing the antigen [131]. A number of clinical trials using cancer vaccines in OC are underway, but no vaccine has been approved for the treatment of OC patients yet [131]. A stage 1, single-arm pilot study testing an IL-17-producing T-cell (Th17) DC vaccine in 19 late-stage OC patients found that the vaccine caused no adverse side effects or toxicity; a proportion of 89% of patients showed significantly higher interferon T-cell responses, and the vaccine was suggested to increase progression-free survival [132]. Interestingly for OC patients with immunologically ‘cold’ tumors, a randomized retrospective phase 2 trial testing debulking surgery with chemotherapy alone or in conjunction with a DC vaccine found that while ‘hot’ tumors benefit from chemotherapy but experience no benefit when combined with DC vaccines, patients with ‘cold’ tumors experienced a significant increase in survival outcomes when the DC vaccine was used with chemotherapy [61]. 

Yet another therapeutic approach to the treatment of OC is the use of oncolytic viruses (OVs). OVs ultimately function to induce the oncolysis of tumor cells, induce an anti-tumor immune response, and cause vascular shutdown in tumors without adversely affecting healthy cells [133]. Between 2013 and 2022, 289 clinical trials have been undertaken, specifically testing the use of various oncolytic viruses on different cancers, often in conjunction with other immunotherapies [134]. In an open-label phase 1 trial using a modified vaccinia virus, some patients experienced a reduction in measurements of tumor size, increased CD8+ T cell infiltration, and an increase in progression-free survival [135]. However, the vascular shutdown induced by OVs can lead to further increases in tumor hypoxia, leading to elevated expression of pro-angiogenic factors such as VEGF and aggressive tumor regrowth [136,137]. OV-induced oncolysis stimulates intratumoral immunity and recruitment of immune cells to migrate to the tumor [138,139]. However, by inducing vascular shutdown, OVs may decrease the uptake of the immune cells recruited to the tumor, inhibiting their anti-tumor efficacy [136]. In a murine model of OC, it was found that the combination of Newcastle disease virus with a molecule that stimulates vascular normalization resulted in enhanced tumor perfusion, reduced tumor size, a decreased number of metastatic tumors, and increased immune cell migration to the tumor [140]. It appears that remodeling the TME to improve perfusion may be a mechanism to increase OV efficacy. 

While immunotherapies show significant potential, the most effective approaches and optimal combination therapies still need to be determined. Immune activation can also have undesirable effects if not managed properly [141]. In a double-blind, phase 3 trial including 945 patients with late-stage melanoma testing the combination of nivolumab (anti-PD-L1 ICI) and ipilimumab (anti CTLA-4 ICI), 55% of patients experienced severe treatment-related side effects [142] compared to 16.3% and 27.3% of patients prescribed nivolumab and ipilimumab alone, respectively [142]. Synergistic effects of immunotherapies, especially ICIs, are not completely understood, and more work needs to be done to avoid any cytokine- or immune-related toxicities that could occur [141].

Aside from immunotherapies, there are targeted therapies in OC that target specific growth factors, receptors, signal transduction pathways, DNA repair mechanisms, and angiogenesis [143]. In OC, Poly-Adenosine Diphosphate Ribose Polymerase inhibitors (PARPis) have been introduced in clinics to preferentially kill cancer cells that have a BRCA mutation. In BRCA-mutated cells, the ability to perform base excision repair is lost. However, single-stranded breaks in these mutated cells can be repaired by DNA repair molecules such as PARP [144]. PARPi therapy acts by inhibiting ribozyme PARP-1, which senses single-strand DNA breaks (SSBs) in mutated cells. PARP binds to the SSBs and releases an ADP-ribose, subsequently recruiting additional DNA repair factors to the site [145]. An SSB left unrepaired can be converted into a double-strand DNA break (DSB), which is lethal for a cell if repaired improperly or left unrepaired [146]. The additive effect of PARPi, along with platinum-based chemotherapy, is being investigated in ongoing clinical trials to determine the optimal use of PARPis [147]. Full approval for the clinical use of PARPis was supported by phase II and III clinical studies involving the use of PARPi olaparib, niraparib, or rucaparib [148]. Pujade-Laurane et al. (2017) performed a phase III double-blind trial examining the survival outcomes of relapsed OC patients who had received at least two rounds of platinum chemotherapy. Of the 195 patients who received olaparib, the median progression-free survival was 19.1 months compared to the placebo median of 5.5 months [149]. A similar phase III study performed by Coleman et al. (2017) found that patients with BRCA-mutant OC had progression-free survival of 16.6 months with the PARP inhibitor compared to 5.4 months with the placebo. It is evident that those with the BRCA mutation may benefit the most from PARPi, and there is promise that administering PARPi therapy in these patients can have significant clinical benefit [150]. Interestingly, PARPis also have significant effects on the TME. PARPis inhibit HIF expression, which facilitates repolarization of M2 macrophages to an M1 phenotype [151]. By inhibiting HIF expression, PARPis also stimulate DC activity and promote B-cell maturation and B-cell memory formation [152,153]. PARPis also decrease the immunosuppression typically seen in solid-tumor TMEs by stimulating CD8+ T cells, increasing IFN expression, and suppressing the activity of Tregs [154].

## 7. Remodeling the TME to Enhance Therapeutic Efficacy

It was originally hypothesized that blocking the formation of new blood vessels to essentially “starve” the tumor of oxygen and nutrients would help keep tumors in a dormant state [155]. Anti-angiogenic strategies have included monoclonal antibodies (mAbs), ligand inhibitors, and tyrosine kinase inhibitors (TKIs), often targeting VEGF [156]. The first anti-angiogenic drug to be approved by the FDA was anti-VEGF mAb bevacizumab (Avastin), which disrupts VEGF signaling to reduce angiogenesis. Its use was approved in 2013 in combination with standard treatments of chemotherapy (carboplatin or paclitaxel) [157]. A number of clinical trials have been conducted on the use of bevacizumab in OC, including a randomized trial of 1873 women with epithelial ovarian, fallopian tube, or primary peritoneal stage III or IV cancer. Progression-free survival (PFS) was 14.1 months, compared to 10.3 months for standard treatment alone [91]. In another phase III clinical trial administering the same dosing schedules, PFS was 19.8 months, compared to 17.4 months for standard treatment [158]. Anti-angiogenic approaches have failed to have the significant anti-tumor effects that were hoped for when they were first developed, and side effects have been problematic. Targeting the VEGF pathway has had deleterious effects of non-specificity and vascularly mediated disorders such as thrombosis and impaired endothelial cell regeneration, resulting in an increased risk of hemorrhage [159].

It is understood that the process of angiogenesis causes barriers to treatment efficacy not only due to immune responses to hypoxia but also the faulty delivery systems of therapeutic drugs and activated immune cells [160]. In contrast, targeting the vasculature through normalization has opened a new strategy for targeted cancer therapies to improve perfusion into the tumor [161]. Clinical trials are underway for developed anti-angiogenic drugs that target tumor vessels by remodeling the vasculature to a healthier morphology. So far, monoclonal antibodies such as vanucizumab with dual-modality mechanisms that target the function of VEGF and angiopentin-2 to modulate the formation of abnormal vessels have been explored [162]. After a phase I trial, Hidalgo et al. (2018) suggested the use of vessel-normalizing therapies in combination with standard chemotherapy and immunotherapies for optimal therapeutic benefits [163]. This idea of combined therapy has brought considerable interest to cancer researchers and patients to not only target the disease more efficiently but also overcome the toxic side effects of standard therapies [164]. Standard chemotherapy in OC is often administered at maximum tolerated doses (MTDs), where patients experience severe negative side effects and co-morbidities and must recover during a drug-free interval [165]. The drug-free interval allows time for cancer cells to develop resistance mechanisms against the chemotherapy’s anti-tumoral actions [166]. Limited perfusion to the tumor is why patients must undergo chemotherapy at the MTD [167]. The mechanism behind this combined therapy suggests that normalizing the vasculature can improve therapeutic uptake and success in OC patients [168].

## 8. Conclusions and Future Directions

The ovarian TME is complex, with numerous features that create therapeutic challenges. As a result of dysfunctional vasculature, high IFP, and widespread immunosuppression, therapeutic efficacy has been limited. It is understood that the immunosuppressive environment seen in the ovarian TME is due to the dysfunction of the tumor vasculature and resultant tissue hypoxia and elevated tumor IFP. Historically, there has been a monotherapeutic approach to treating OC, often using either chemotherapy or immunotherapy. Going forward, the use of combination therapy to combat several characteristics of the TME will be needed. Up-front remodeling of the TME, such as through the use of vascular normalization, can make the tumor more tolerant of therapies and could make tumor cells more responsive to other secondary therapies. With this approach, we must not only be concerned about what drugs are used in combination but also about the timing of administration of these compounds. With further understanding of the ovarian TME, there are approaches being developed to remodel the microenvironment to enhance the uptake and efficacy of therapies such as cytotoxic chemotherapy, oncolytic viruses, and immunotherapies. The development of novel therapies, in conjunction with TME remodeling, can result in impactful approaches for this disease for which improvements in therapeutic response have not occurred for decades.

## Figures and Tables

**Table 1:** Targeted therapies and small-molecule inhibitors in ovarian cancer.

Inhibitor	Target	Effect	Reference
miR-204-5p-inh	miR-204-5p	Inhibits angiogenesis and ovarian tumor growth	[95]
TW-37	Bcl-2	Apoptosis of ovarian cancer cells; resensitization to cisplatin	[96]
SC144	Glycoprotein 130 (gp130)	Delays ovarian tumor progression	[97]
PACMA 31	Protein Disulfide Isomerase (PDI)	Reduces survival and proliferation of ovarian cancer cells; delays tumor growth	[98]
TAK242	Toll-like receptor (TLR) 4	Suppression of ovarian cancer cell inflammation; induction of cell cycle arrest and apoptosis	[99]
Bevacizumab	VEGF	Has shown clinical efficacy, particularly when used in combination	[100]
Cetuximab	EGFR	Reduces cell proliferation and inhibits cell cycle	[101]
Pertuzumab	HER2	Targets HER2 dimerization domain—inhibits HER signaling and reduces cell proliferation	[101]
Sorafenib	Multi-receptor tyrosine kinases	Cytotoxic to tumor cells; anti-angiogenic	[102]
Erlotinib	EGFR	Can reduce ovarian cancer progression in EGFR-positive tumors	[103]
Olaparib	PARP	Synthetic lethality in ovarian tumors; enhanced efficacy in patients with BRCA mutations or homologous recombination-deficient tumors	[104]
Niraparib	PARP	Synthetic lethality; efficacy appears not to be reliant on BRCA status	[105]
Pembrolizumab	PD-1	Reduces tumor immunosuppression; reduced tumor progression in clinical trials	[106]
Ipilimumab	CTLA-4	Enhances expansion of tumor-infiltrating lymphocytes in the ovarian TME	[107]
Durvalumab	PD-L1	Reduces immunosuppression in the ovarian TME	[108]

## Data Availability

Not applicable.

## References

[1] Peres L.C., Cushing-Haugen K.L., Kobel M., Harris H.R., Berchuck A., Rossing M.A., Schildkraut J.M., A Doherty J. (2019). Invasive Epithelial Ovarian Cancer Survival by Histotype and Disease Stage. J. Natl. Cancer Inst..

[2] Jayson G.C., Kohn E.C., Kitchener H.C., Ledermann J.A. (2014). Ovarian cancer. Lancet.

[3] Arora T., Mullangi S., Lekkala M.R. (2024). Ovarian Cancer. StatPearls.

[4] Valkenburg K.C., de Groot A.E., Pienta K.J. (2018). Targeting the tumour stroma to improve cancer therapy. Nat. Rev. Clin. Oncol..

[5] Henke E., Nandigama R., Ergün S. (2019). Extracellular Matrix in the Tumor Microenvironment and Its Impact on Cancer Therapy. Front. Mol. Biosci..

[6] Hynes R.O., Naba A. (2012). Overview of the matrisome—An inventory of extracellular matrix constituents and functions. Cold Spring Harb. Perspect. Biol..

[7] Walker C., Mojares E., del Río Hernández A. (2018). Role of Extracellular Matrix in Development and Cancer Progression. Int. J. Mol. Sci..

[8] Radisky E.S. (2024). Extracellular proteolysis in cancer: Proteases, substrates, and mechanisms in tumor progression and metastasis. J. Biol. Chem..

[9] Amos S.E., Choi Y.S. (2021). The Cancer Microenvironment: Mechanical Challenges of the Metastatic Cascade. Front. Bioeng. Biotechnol..

[10] Sleeboom J.J.F., van Tienderen G.S., Schenke-Layland K., van der Laan L.J.W., Khalil A.A., Verstegen M.M.A. (2024). The extracellular matrix as hallmark of cancer and metastasis: From biomechanics to therapeutic targets. Sci. Transl. Med..

[11] Lampi M.C., Reinhart-King C.A. (2018). Targeting extracellular matrix stiffness to attenuate disease: From molecular mechanisms to clinical trials. Sci. Transl. Med..

[12] Labiche A., Heutte N., Herlin P., Chasle J., Gauduchon P., Elie N. (2010). Stromal compartment as a survival prognostic factor in advanced ovarian carcinoma. Int. J. Gynecol. Cancer.

[13] Joshi R.S., Kanugula S.S., Sudhir S., Pereira M.P., Jain S., Aghi M.K. (2021). The Role of Cancer-Associated Fibroblasts in Tumor Progression. Cancers.

[14] Rodriguez G.M., Galpin K.J.C., McCloskey C.W., Vanderhyden B.C. (2018). The Tumor Microenvironment of Epithelial Ovarian Cancer and Its Influence on Response to Immunotherapy. Cancers.

[15] Xing F., Saidou J., Watabe K. (2010). Cancer associated fibroblasts (CAFs) in tumor microenvironment. Front. Biosci. Landmark Ed..

[16] Desmoulière A., Redard M., Darby I., Gabbiani G. (1995). Apoptosis mediates the decrease in cellularity during the transition between granulation tissue and scar. Am. J. Pathol..

[17] Bremnes R.M., Dønnem T., Al-Saad S., Al-Shibli K., Andersen S., Sirera R., Camps C., Marinez I., Busund L.-T. (2011). The role of tumor stroma in cancer progression and prognosis: Emphasis on carcinoma-associated fibroblasts and non-small cell lung cancer. J. Thorac. Oncol..

[18] Helms E., Onate M.K., Sherman M.H. (2020). Fibroblast Heterogeneity in the Pancreatic Tumor Microenvironment. Cancer Discov..

[19] Davidson B., Trope C.G., Reich R. (2014). The role of the tumor stroma in ovarian cancer. Front. Oncol..

[20] Kamat A.A., Fletcher M., Gruman L.M., Mueller P., Lopez A., Landen C.N., Han L., Gershenson D.M., Sood A.K. (2006). The clinical relevance of stromal matrix metalloproteinase expression in ovarian cancer. Clin. Cancer Res..

[21] Luo Z., Wang Q., Lau W.B., Lau B., Xu L., Zhao L., Yang H., Feng M., Xuan Y., Yang Y. (2016). Tumor microenvironment: The culprit for ovarian cancer metastasis?. Cancer Lett..

[22] Bamberger E.S., Perrett C.W. (2002). Angiogenesis in epithelian ovarian cancer. Mol. Pathol..

[23] Teleanu R.I., Chircov C., Grumezescu A.M., Teleanu D.M. (2019). Tumor Angiogenesis and Anti-Angiogenic Strategies for Cancer Treatment. J. Clin. Med..

[24] Joyce J.A., Baruch A., Chehade K., Meyer-Morse N., Giraudo E., Tsai F.Y., Greenbaum D.C., Hager J.H., Bogyo M., Hanahan D. (2004). Cathepsin cysteine proteases are effectors of invasive growth and angiogenesis during multistage tumorigenesis. Cancer Cell.

[25] Deng T., Lu X., Jia X., Du J., Wang L., Cao B., Yang M., Yin Y., Liu F. (2024). Cathepsins and cancer risk: A Mendelian randomization study. Front. Endocrinol..

[26] Pranjol M.Z.I., Gutowski N.J., Hannemann M., Whatmore J.L. (2019). Cathepsin L Induces Proangiogenic Changes in Human Omental Microvascular Endothelial Cells via Activation of the ERK1/2 Pathway. Curr. Cancer Drug Targets.

[27] Pranjol Z.I., Zinovkin D.A., Maskell A.R.T., Stephens L.J., Achinovich S.L., Los’ D.M., Nadyrov E.A., Hannemann M., Gutowski N.J., Whatmore J.L. (2019). Cathepsin L-induced galectin-1 may act as a proangiogenic factor in the metastasis of high-grade serous carcinoma. J. Transl. Med..

[28] Hu L., Roth J.M., Brooks P., Luty J., Karpatkin S. (2008). Thrombin up-regulates cathepsin D which enhances angio-genesis, growth, and metastasis. Cancer Res..

[29] Briozzo P., Badet J., Capony F., Pieri I., Montcourrier P., Barritault D., Rochefort H. (1991). MCF7 mammary cancer cells respond to bFGF and internalize it following its release from extracellular matrix: A permissive role of cathepsin D. Exp. Cell Res..

[30] Mustafa A., Elkhamisy F., Arghiani N., Pranjol M.Z.I. (2023). Potential crosstalk between pericytes and cathepsins in the tumour microenvironment. Biomed. Pharmacother..

[31] Lugano R., Ramachandran M., Dimberg A. (2020). Tumor angiogenesis: Causes, consequences, challenges and opportunities. Cell. Mol. Life Sci..

[32] Winkler J., Abisoye-Ogunniyan A., Metcalf K.J., Werb Z. (2020). Concepts of extracellular matrix remodelling in tumour progression and metastasis. Nat. Commun..

[33] Attane C., Muller C. (2020). Drilling for Oil: Tumor-Surrounding Adipocytes Fueling Cancer. Trends Cancer.

[34] Kimura H., Braun R.D., Ong E.T., Hsu R., Secomb T.W., Papahadjopoulos D., Hong K., Dewhirst M.W. (1996). Fluctuations in red cell flux in tumor microvessels can lead to transient hypoxia and reoxygenation in tumor parenchyma. Cancer Res..

[35] Hashizume H., Baluk P., Morikawa S., McLean J.W., Thurston G., Roberge S., Jain R.K., McDonald D.M. (2000). Openings between defective endothelial cells explain tumor vessel leakiness. Am. J. Pathol..

[36] Yu P., Wang Y., Yuan D., Sun Y., Qin S., Li T. (2023). Vascular normalization: Reshaping the tumor microenvironment and augmenting antitumor immunity for ovarian cancer. Front. Immunol..

[37] Vander Heiden M.G., Cantley L.C., Thompson C.B. (2009). Understanding the Warburg effect: The metabolic require-ments of cell proliferation. Science.

[38] Zhang C., Liu N. (2022). Noncoding RNAs in the Glycolysis of Ovarian Cancer. Front. Pharmacol..

[39] Bogdanov A., Bogdanov A., Chubenko V., Volkov N., Moiseenko F., Moiseyenko V. (2022). Tumor acidity: From hallmark of cancer to target of treatment. Front. Oncol..

[40] Andreucci E., Peppicelli S., Ruzzolini J., Bianchini F., Biagioni A., Papucci L., Magnelli L., Mazzanti B., Stecca B., Calorini L. (2020). The acidic tumor microenvironment drives a stem-like phenotype in melanoma cells. J. Mol. Med..

[41] Da Silva A.C., Jammal M.P., Crispim P.C.A., Murta E.F.C., Nomelini R.S. (2020). The Role of Stroma in Ovarian Cancer. Immunol. Investig..

[42] Nowak M., Klink M. (2020). The Role of Tumor-Associated Macrophages in the Progression and Chemoresistance of Ovarian Cancer. Cells.

[43] Solinas G., Germano G., Mantovani A., Allavena P. (2009). Tumor-associated macrophages (TAM) as major players of the cancer-related inflammation. J. Leukoc. Biol..

[44] Larionova I., Cherdyntseva N., Liu T., Patysheva M., Rakina M., Kzhyshkowska J. (2019). Interaction of tu-mor-associated macrophages and cancer chemotherapy. Oncoimmunology.

[45] Plikus M.V., Wang X., Sinha S., Forte E., Thompson S.M., Herzog E.L., Driskell R.R., Rosenthal N., Biernaskie J., Horsley V. (2021). Fibroblasts: Origins, definitions, and functions in health and disease. Cell.

[46] Cirri P., Chiarugi P. (2011). Cancer associated fibroblasts: The dark side of the coin. Am. J. Cancer Res..

[47] Wright K., Ly T., Kriet M., Czirok A., Thomas S.M. (2023). Cancer-Associated Fibroblasts: Master Tumor Microenvironment Modifiers. Cancers.

[48] Sun W., Fu S. (2019). Role of cancer-associated fibroblasts in tumor structure, composition and the microenvironment in ovarian cancer. Oncol. Lett..

[49] Fujisawa M., Moh-Moh-Aung A., Zeng Z., Yoshimura T., Wani Y., Matsukawa A. (2018). Ovarian stromal cells as a source of cancer-associated fibroblasts in human epithelial ovarian cancer: A histopathological study. PLoS ONE.

[50] Barrett R.L., Pure E. (2020). Cancer-associated fibroblasts an their influence on tumor immunity and immunotherapy. Elife.

[51] Elyada E., Bolisetty M., Laise P., Flynn W.F., Courtois E.T., Burkhart R.A., Teinor J.A., Belleau P., Biffi G., Lucito M.S. (2019). Cross-Species Single-Cell Analysis of Pancreatic Ductal Adenocarcinoma Reveals Antigen-Presenting Cancer-Associated Fibroblasts. Cancer Discov..

[52] Zheng X., Turkowski K., Mora J., Brüne B., Seeger W., Weigert A., Savai R. (2017). Redirecting tumor-associated macrophages to become tumoricidal effectors as a novel strategy for cancer therapy. Oncotarget.

[53] Comito G., Giannoni E., Segura C.P., Barcellos-De-Souza P., Raspollini M.R., Baroni G., Lanciotti M., Serni S., Chiarugi P. (2014). Cancer-associated fibroblasts and M2-polarized macrophages synergize during prostate carcinoma progression. Oncogene.

[54] Monteran L., Erez N. (2019). The Dark Side of Fibroblasts: Cancer-Associated Fibroblasts as Mediators of Immunosuppression in the Tumor Microenvironment. Front. Immunol..

[55] Schreiber R.D., Old L.J., Smyth M.J. (2011). Cancer immunoediting: Integrating immunity’s roles in cancer suppression and promotion. Science.

[56] Zhang L., Conejo-Garcia J.R., Katsaros D., Gimotty P.A., Massobrio M., Regnani G., Makrigiannakis A., Gray H., Schlienger K., Liebman M.N. (2003). Intratumoral T cells, recurrence, and survival in epithelial ovarian cancer. N. Engl. J. Med..

[57] Knutson K.L., Lu H., Stone B., Reiman J.M., Behrens M.D., Prosperi C.M., Gad E.A., Smorlesi A., Disis M.L. (2006). Immunoediting of Cancers May Lead to Epithelial to Mesenchymal Transition. Front. Immunol..

[58] Garnier L., Gkountidi A.-O., Hugues S. (2019). Tumor-Associated Lymphatic Vessel Features and Immunomodulatory Functions. Front. Immunol..

[59] Koukourakis M.I., Giatromanolaki A. (2022). Tumor draining lymph nodes, immune response, and radiotherapy: Towards a revisal of therapeutic principles. Biochim. Biophys. Acta Rev. Cancer.

[60] Stylianopoulos T., Martin J.D., Snuderl M., Mpekris F., Jain S.R., Jain R.K. (2013). Coevolution of solid stress and in-terstitial fluid pressure in tumors during progression: Implications for vascular collapse. Cancer Res..

[61] Fucikova J., Hensler M., Kasikova L., Lanickova T., Pasulka J., Rakova J., Drozenova J., Fredriksen T., Hraska M., Hrnciarova T. (2022). An Autologous Dendritic Cell Vaccine Promotes Anticancer Immunity in Patients with Ovarian Cancer with Low Mutational Burden and Cold Tumors. Clin. Cancer Res..

[62] Fanale D., Dimino A., Pedone E., Brando C., Corsini L.R., Filorizzo C., Fiorino A., Lisanti M.C., Magrin L., Randazzo U. (2022). Prognostic and Predictive Role of Tumor-Infiltrating Lymphocytes (TILs) in Ovarian Cancer. Cancers.

[63] Wu J.W.Y., Dand S., Doig L., Papenfuss A.T., Scott C.L., Ho G., Ooi J.D. (2021). T-Cell Receptor Therapy in the Treatment of Ovarian Cancer: A Mini Review. Front. Immunol..

[64] Tang S., Ning Q., Yang L., Mo Z., Tang S. (2020). Mechanisms of immune escape in the cancer immune cycle. Int. Immunopharmacol..

[65] Vinay D.S., Ryan E.P., Pawelec G., Talib W.H., Stagg J., Elkord E., Lichtor T., Decker W.K., Whelan R.L., Kumara H.M.C.S. (2015). Immune evasion in cancer: Mechanistic basis and therapeutic strategies. Seminars in Cancer Biology.

[66] Pan Y., Yu Y., Wang X., Zhang T. (2020). Tumor-Associated Macrophages in Tumor Immunity. Front. Immunol..

[67] Wei C., Liu X., Wang Q., Li Q., Xie M. (2021). Identification of Hypoxia Signature to Assess the Tumor Immune Microenvironment and Predict Prognosis in Patients with Ovarian Cancer. Int. J. Endocrinol..

[68] Jing X., Yang F., Shao C., Wei K., Xie M., Shen H., Shu Y. (2019). Role of hypoxia in cancer therapy by regulating the tumor microenvironment. Mol. Cancer.

[69] Noman M.Z., Desantis G., Janji B., Hasmim M., Karray S., Dessen P., Bronte V., Chouaib S. (2014). PD-L1 is a novel direct target of HIF-1α, and its blockade under hypoxia enhanced MDSC-mediated T cell activation. J. Exp. Med..

[70] Yu J., Zhuang A., Gu X., Hua Y., Yang L., Ge S., Ruan J., Chai P., Jia R., Fan X. (2023). Nuclear PD-L1 promotes EGR1-mediated angiogenesis and accelerates tumorigenesis. Cell Discov..

[71] Yu W., Hua Y., Qiu H., Hao J., Zou K., Li Z., Hu S., Guo P., Chen M., Sui S. (2020). PD-L1 promotes tumor growth and progression by activating WIP and beta-catenin signaling pathways and predicts poor prognosis in lung cancer. Cell Death Dis..

[72] Barsoum I.B., Smallwood C.A., Siemens D.R., Graham C.H. (2014). A mechanism of hypoxia-mediated escape from adaptive immunity in cancer cells. Cancer Res..

[73] Koh Y.W., Lee S.J., Han J.H., Haam S., Jung J., Lee H.W. (2019). PD-L1 protein expression in non-small-cell lung cancer and its relationship with the hypoxia-related signaling pathways: A study based on immunohistochemistry and RNA sequencing data. Lung Cancer.

[74] Facciabene A., Peng X., Hagemann I.S., Balint K., Barchetti A., Wang L.P., Gimotty P.A., Gilks C.B., Lal P., Zhang L. (2011). Tumour hypoxia promotes tolerance and angiogenesis via CCL28 and T(reg) cells. Nature.

[75] Kryczek I., Wei S., Zhu G., Myers L., Mottram P., Cheng P., Chen L., Coukos G., Zou W. (2007). Relationship between B7-H4, regulatory T cells, and patient outcome in human ovarian carcinoma. Cancer Res..

[76] Curiel T.J., Coukos G., Zou L., Alvarez X., Cheng P., Mottram P., Evdemon-Hogan M., Conejo-Garcia J.R., Zhang L., Burow M. (2004). Specific recruitment of regulatory T cells in ovarian carcinoma fosters immune privilege and predicts reduced survival. Nat. Med..

[77] Dufait I., Pardo J., Escors D., De Vlaeminck Y., Jiang H., Keyaerts M., De Ridder M., Breckpot K. (2019). Perforin and Granzyme B Expressed by Murine Myeloid-Derived Suppressor Cells: A Study on Their Role in Outgrowth of Cancer Cells. Cancers.

[78] Saleh R., Elkord E. (2020). FoxP3+ T regulatory cells in cancer: Prognostic biomarkers and therapeutic targets. Cancer Lett..

[79] Buchbinder E.I., Desai A. (2016). CTLA-4 and PD-1 Pathways: Similarities, Differences, and Implications of Their inhibition. Am. J. Clin. Oncol..

[80] Zeng Y., Li B., Liang Y., Reeves P.M., Qu X., Ran C., Liu Q., Callahan M.V., Sluder A.E., Gelfand J.A. (2019). Dual blockade of CXCL12-CXCR4 and PD-1-PD-L1 pathways prolongs survival of ovarian tumor-bearing mice by prevention of immunosuppression in the tumor microenvironment. FASEB J..

[81] Chou F.-C., Chen H.-Y., Kuo C.-C., Sytwu H.-K. (2018). Role of Galectins in Tumors and in Clinical Immunotherapy. Int. J. Mol. Sci..

[82] Astorgues-Xerri L., Riveiro M.E., Tijeras-Raballand A., Serova M., Neuzillet C., Albert S., Raymond E., Faivre S. (2014). Unraveling galectin-1 as a novel therapeutic target for cancer. Cancer Treat. Rev..

[83] Cagnoni A.J., Giribaldi M.L., Blidner A.G., Cutine A.M., Gatto S.G., Morales R.M., Salatino M., Abba M.C., Croci D.O., Mariño K.V. (2021). Galectin-1 fosters an immunosuppressive microenvironment in colorectal cancer by reprogramming CD8^+^ regulatory T cells. Proc. Natl. Acad. Sci. USA.

[84] Yakushina V.D., Vasil’eva O.A., Ryazantseva N.V., Novitsky V.V., Tashireva L.A. (2015). The effects of galectin-1 on the gene expression of the transcription factors TBX21, GATA-3, FOXP3 and RORC. Mol. Cell. Biochem..

[85] Schweer D., McAtee A., Neupane K., Richards C., Ueland F., Kolesar J. (2022). Tumor-Associated Macrophages and Ovarian Cancer: Implications for Therapy. Cancers.

[86] Cheng H., Wang Z., Fu L., Xu T. (2019). Macrophage Polarization in the Development and Progression of Ovarian Cancers: An Overview. Front. Oncol..

[87] Zhang Q., Cai D.-J., Li B. (2015). Ovarian cancer stem-like cells elicit the polarization of M2 macrophages. Mol. Med. Rep..

[88] Zhang M., He Y., Sun X., Li Q., Wang W., Zhao A., Di W. (2014). A high M1/M2 ratio of tumor-associated macrophages is associated with extended survival in ovarian cancer patients. J. Ovarian Res..

[89] Lan C., Huang X., Lin S., Huang H., Cai Q., Wan T., Lu J., Liu J. (2013). Expression of M2-polarized macrophages is associated with poor prognosis for advanced epithelial ovarian cancer. Technol. Cancer Res. Treat..

[90] Yuan X., Zhang J., Li D., Mao Y., Mo F., Du W., Ma X. (2017). Prognostic significance of tumor-associated macrophages in ovarian cancer: A meta-analysis. Gynecol. Oncol..

[91] Tiwari A., Trivedi R., Lin S.-Y. (2022). Tumor microenvironment: Barrier or opportunity towards effective cancer therapy. J. Biomed. Sci..

[92] Gonzalez H., Hagerling C., Werb Z. (2018). Roles of the immune system in cancer: From tumor initiation to metastatic progression. Genes Dev..

[93] Zhao H., Wu L., Yan G., Chen Y., Zhou M., Wu Y., Li Y. (2021). Inflammation and tumor progression: Signaling pathways and targeted intervention. Signal Transduct. Target. Ther..

[94] Khalaf K., Hana D., Chou J.T., Singh C., Mackiewicz A., Kaczmarek M. (2021). Aspects of the Tumor Microenviron-ment Involved in Immune Resistance and Drug Resistance. Front. Immunol..

[95] Chen X., Mangala L.S., Mooberry L., Bayraktar E., Dasari S.K., Ma S., Ivan C., Court K.A., Rodriguez-Aguayo C., Bayraktar R. (2019). Identifying and targeting angiogenesis-Related microRNAs in ovarian cancer. Oncogene.

[96] Wang H., Zhang Z., Wei X., Dai R. (2015). Small-molecule inhibitor of Bcl-2 (TW-37) suppresses growth and enhances cisplatin-induced apoptosis in ovarian cancer cells. J. Ovarian Res..

[97] Xu S., Grande F., Garofalo A., Neamati N., Gu D., Liu H., Su G.H., Zhang X., Chin-Sinex H., Hanenberg H. (2013). Discovery of a novel orally active small-molecule gp130 inhibitor for the treatment of ovarian cancer. Mol. Cancer Ther..

[98] Xu S., Butkevich A.N., Yamada R., Zhou Y., Debnath B., Duncan R., Zandi E., Petasis N.A., Neamati N. (2012). Discovery of an orally active small-molecule irreversible inhibitor of protein disulfide isomerase for ovarian cancer treatment. Proc. Natl. Acad. Sci. USA.

[99] Kashani B., Zandi Z., Bashash D., Zaghal A., Momeny M., Poursani E.M., Pourbagheri-Sigaroodi A., Mousavi S.A., Ghaffari S.H. (2020). Small molecule inhibitor of TLR4 inhibits ovarian cancer cell proliferation: New insight into the anticancer effect of TAK-242 (Resatorvid). Cancer Chemother. Pharmacol..

[100] Xie Y., Zhou F. (2024). Efficacy and safety of anti-angiogenic drug monotherapy and combination therapy for ovarian cancer: A meta-analysis and trial sequential analysis of randomized controlled trials. Front. Pharmacol..

[101] Rendell A., Thomas-Bland I., McCuish L., Taylor C., Binju M., Yu Y. (2022). Targeting Tyrosine Kinases in Ovarian Cancer: Small Molecule Inhibitor and Monoclonal Antibody, Where Are We Now?. Biomedicines.

[102] Hainsworth J.D., Thompson D.S., Bismayer J.A., Gian V.G., Merritt W.M., Whorf R.C., Finney L.H., Dudley B.S. (2015). Paclitaxel/carboplatin with or without sorafenib in the first-line treatment of patients with stage III/IV epithelial ovarian cancer: A randomized phase II study of the Sarah Cannon Research Institute. Cancer Med..

[103] Despierre E., Vergote I., Anderson R., Coens C., Katsaros D., Hirsch F.R., Boeckx B., Varella-Garcia M., Ferrero A., Ray-Coquard I. (2015). Epidermal Growth Factor Receptor (EGFR) Pathway Biomarkers in the Randomized Phase III Trial of Erlotinib Versus Observation in Ovarian Cancer Patients with No Evidence of Disease Progression after First-Line Platinum-Based Chemotherapy. Target. Oncol..

[104] Lheureux S., Prokopec S.D., Oldfield L.E., Gonzalez-Ochoa E., Bruce J.P., Wong D., Danesh A., Torti D., Torchia J., Fortuna A. (2023). Identifying Mechanisms of Resistance by Circulating Tumor DNA in EVOLVE, a Phase II Trial of Cediranib Plus Olaparib for Ovarian Cancer at Time of PARP Inhibitor Progression. Clin. Cancer Res..

[105] Gonzalez-Martin A., Pothuri B., Vergote I., DePont Christensen R., Graybill W., Mirza M.R., McCormick C., Lorusso D., Hoskins P., Freyer G. (2019). Niraparib in Patients with Newly Diagnosed Advanced Ovarian Cancer. N. Engl. J. Med..

[106] Matulonis U.A., Shapira-Frommer R., Santin A.D., Lisyanskaya A.S., Pignata S., Vergote I., Raspagliesi F., Sonke G.S., Birrer M., Provencher D.M. (2019). Antitumor activity and safety of pembrolizumab in patients with advanced re-current ovarian cancer: Results from the phase II KEYNOTE-100 study. Ann. Oncol..

[107] Kverneland A.H., Pedersen M., Westergaard M.C.W., Nielsen M., Borch T.H., Olsen L.R., Aasbjerg G., Santegoets S.J., van der Burg S.H., Milne K. (2020). Adoptive cell therapy in combination with checkpoint inhibitors in ovarian cancer. Oncotarget.

[108] Farokhi Boroujeni S., Rodriguez G., Galpin K., Yakubovich E., Murshed H., Ibrahim D., Asif S., Vanderhyden B.C. (2023). BRCA1 and BRCA2 deficient tumour models generate distinct ovarian tumour microenvironments and differential responses to therapy. J. Ovarian Res..

[109] Feng B., Zhou F., Hou B., Wang D., Wang T., Fu Y., Ma Y., Yu H., Li Y. (2018). Binary Cooperative Prodrug Nano-particles Improve Immunotherapy by Synergistically Modulating Immune Tumor Microenvironment. Adv. Mater..

[110] Raja F.A., Chopra N., Ledermann J.A. (2012). Optimal first-line treatment in ovarian cancer. Ann. Oncol..

[111] Ventola C.L. (2017). Cancer Immunotherapy, Part 3: Challenges and Future Trends. Pharm. Ther..

[112] Yang C., Xia B.-R., Zhang Z.-C., Zhang Y.-J., Lou G., Jin W.-L. (2020). Immunotherapy for Ovarian Cancer: Adjuvant, Combination, and Neoadjuvant. Front. Immunol..

[113] Johnson R.L., Cummings M., Thangavelu A., Theophilou G., de Jong D., Orsi N.M. (2021). Barriers to Immunotherapy in Ovarian Cancer: Metabolic, Genomic, and Immune Perturbations in the Tumour Microenvironment. Cancers.

[114] McFarland C.D., Yaglom J.A., Wojtkowiak J.W., Scott J.G., Morse D.L., Sherman M.Y., Mirny L.A. (2017). The Dam-aging Effect of Passenger Mutations on Cancer Progression. Cancer Res..

[115] Rad H.S., Monkman J., Warkiani M.E., Ladwa R., O’Byrne K., Rezaei N., Kulasinghe A. (2021). Understanding the tumor microenvironment for effective immunotherapy. Med. Res. Rev..

[116] Morand S., Devanaboyina M., Staats H., Stanbery L., Nemunaitis J. (2021). Ovarian Cancer Immunotherapy and Personalized Medicine. Int. J. Mol. Sci..

[117] Palaia I., Tomao F., Sassu C.M., Musacchio L., Benedetti Panici P. (2020). Immunotherapy For Ovarian Cancer: Recent Advances And Combination Therapeutic Approaches. OncoTargets Ther..

[118] Muthukutty P., Woo H.Y., Ragothaman M., Yoo S.Y. (2023). Recent Advances in Cancer Immunotherapy Delivery Modalities. Pharmaceutics.

[119] Cunningham N., Lapointe R., Lerouge S. (2022). Biomaterials for enhanced immunotherapy. APL Bioeng..

[120] Sun Q., Hong Z., Zhang C., Wang L., Han Z., Ma D. (2023). Immune checkpoint therapy for solid tumours: Clinical dilemmas and future trends. Signal Transduct. Target. Ther..

[121] Blanc-Durand F., Genestie C., Galende E.Y., Gouy S., Morice P., Pautier P., Maulard A., Mesnage S., Le Formal A., Brizais C. (2021). Distribution of novel immune-checkpoint targets in ovarian cancer tumor microenvironment: A dynamic landscape. Gynecol. Oncol..

[122] Lampert E.J., Zimmer A.S., Padget M.R., Cimino-Mathews A., Nair J.R., Liu Y., Swisher E.M., Hodge J.W., Nixon A.B., Nichols E. (2020). Combination of PARP Inhibitor Olaparib, and PD-L1 Inhibitor Durvalumab, in Recurrent Ovarian Cancer: A Proof-of-Concept Phase II Study. Clin. Cancer Res..

[123] Schoutrop E., El-Serafi I., Poiret T., Zhao Y., Gultekin O., He R., Moyano-Galceran L., Carlson J.W., Lehti K., Hassan M. (2021). Mesothelin-Specific CAR T Cells Target Ovarian Cancer. Cancer Res..

[124] Xia A.-L., Wang X.-C., Lu Y.-J., Lu X.-J., Sun B. (2017). Chimeric-antigen receptor T (CAR-T) cell therapy for solid tumors: Challenges and opportunities. Oncotarget.

[125] Dong X., Ren J., Amoozgar Z., Lee S., Datta M., Roberge S., Duquette M., Fukumura D., Jain R.K. (2023). Anti-VEGF therapy improves EGFR-vIII-CAR-T cell delivery and efficacy in syngeneic glioblastoma models in mice. J. ImmunoTherapy Cancer.

[126] Kang C., Jeong S.-Y., Song S.Y., Choi E.K. (2020). The emerging role of myeloid-derived suppressor cells in radiotherapy. Radiat. Oncol. J..

[127] Pegram H.J., Lee J.C., Hayman E.G., Imperato G.H., Tedder T.F., Sadelain M., Brentjens R.J. (2012). Tumor-targeted T cells modified to secrete IL-12 eradicate systemic tumors without need for prior conditioning. Blood.

[128] Krenciute G., Prinzing B.L., Yi Z., Wu M.F., Liu H., Dotti G., Balyasnikova I.V., Gottschalk S. (2017). Transgenic Ex-pression of IL15 Improves Antiglioma Activity of IL13Ralpha2-CAR T Cells but Results in Antigen Loss Variants. Cancer Immunol. Res..

[129] Spolski R., Leonard W.J. (2014). Interleukin-21: A double-edged sword with therapeutic potential. Nat. Rev. Drug Discov..

[130] Shimabukuro-Vornhagen A., Godel P., Subklewe M., Stemmler H.J., Schlosser H.A., Schlaak M., Kochanek M., Boll B., von Bergwelt-Baildon M.S. (2018). Cytokine release syndrome. J. ImmunoTherapy Cancer.

[131] Chow S., Berek J.S., Dorigo O. (2020). Development of Therapeutic Vaccines for Ovarian Cancer. Vaccines.

[132] Block M.S., Dietz A.B., Gustafson M.P., Kalli K.R., Erskine C.L., Youssef B., Vijay G.V., Allred J.B., Pavelko K.D., Strausbauch M.A. (2020). Th17-inducing autologous dendritic cell vaccination promotes antigen-specific cellular and humoral immunity in ovarian cancer patients. Nat. Commun..

[133] Kooti W., Esmaeili Gouvarchin Ghaleh H., Farzanehpour M., Dorostkar R., Jalali Kondori B., Bolandian M. (2021). Oncolytic Viruses and Cancer, Do You Know the Main Mechanism?. Front. Oncol..

[134] Yun C.-O., Hong J., Yoon A.-R. (2022). Current clinical landscape of oncolytic viruses as novel cancer immunotherapeutic and recent preclinical advancements. Front. Immunol..

[135] Manyam M., Stephens A.J., Kennard J.A., LeBlanc J., Ahmad S., Kendrick J.E., Holloway R.W. (2021). A phase 1b study of intraperitoneal oncolytic viral immunotherapy in platinum-resistant or refractory ovarian cancer. Gynecol. Oncol..

[136] Santry L.A., van Vloten J.P., Knapp J.P., Matuszewska K., McAusland T.M., Minott J.A., Mould R.C., Stegelmeier A.A., Major P.P., Wootton S.K. (2020). Tumour vasculature: Friend or foe of oncolytic viruses?. Cytokine Growth Factor Rev..

[137] Beug S.T., Pichette S.J., St-Jean M., Holbrook J., Walker D.E., LaCasse E.C., Korneluk R.G. (2018). Combination of IAP Antagonists and TNF-α-Armed Oncolytic Viruses Induce Tumor Vascular Shutdown and Tumor Regression. Mol. Ther. Oncol..

[138] Fu R., Qi R., Xiong H., Lei X., Jiang Y., He J., Chen F., Zhang L., Qiu D., Chen Y. (2024). Combination therapy with oncolytic virus and T cells or mRNA vaccine amplifies antitumor effects. Signal Transduct. Target. Ther..

[139] Santry L.A., van Vloten J.P., AuYeung A.W.K., Mould R.C., Yates J.G.E., McAusland T.M., Petrik J.J., Major P.P., Bridle B.W., Wootton S.K. (2024). Recombinant Newcastle disease viruses expressing immunological checkpoint inhibitors induce a pro-inflammatory state and enhance tumor-specific immune responses in two murine models of cancer. Front. Microbiol..

[140] Matuszewska K., Santry L.A., van Vloten J.P., Auyeung A.W.K., Major P.P., Lawler J., Wootton S.K., Bridle B.W., Petrik J. (2019). Combining Vascular Normalization with an Oncolytic Virus Enhances Immunotherapy in a Preclinical Model of Advanced-Stage Ovarian Cancer. Clin. Cancer Res..

[141] Lynam S., Lugade A.A., Odunsi K. (2020). Immunotherapy for Gynecologic Cancer: Current Applications and Future Directions. Clin. Obstet. Gynecol..

[142] Larkin J., Chiarion-Sileni V., Gonzalez R., Grob J.-J., Rutkowski P., Lao C.D., Cowey C.L., Schadendorf D., Wagstaff J., Dummer R. (2019). Five-Year Survival with Combined Nivolumab and Ipilimumab in Advanced Melanoma. N. Engl. J. Med..

[143] Shuel S.L. (2022). Targeted cancer therapies: Clinical pearls for primary care. Can. Fam. Physician.

[144] Turk A.A., Wisinski K.B. (2018). PARP inhibitors in breast cancer: Bringing synthetic lethality to the bedside. Cancer.

[145] Wu Y., Xu S., Cheng S., Yang J., Wang Y. (2023). Clinical application of PARP inhibitors in ovarian cancer: From mo-lecular mechanisms to the current status. J. Ovarian Res..

[146] Boussios S., Rassy E., Moschetta M., Ghose A., Adeleke S., Sanchez E., Sheriff M., Chargari C., Pavlidis N. (2022). BRCA Mutations in Ovarian and Prostate Cancer: Bench to Bedside. Cancers.

[147] Rose P.G. (2022). Ovarian cancer recurrence: Is the definition of platinum sensitivity modified by PARPi, bevacizumab or other intervening treatments?: A clinical perspective. Cancer Drug Resist..

[148] Konstantinopoulos P.A., Lheureux S., Moore K.N. (2020). PARP Inhibitors for Ovarian Cancer: Current Indications, Future Combinations, and Novel Assets in Development to Target DNA Damage Repair. Am. Soc. Clin. Oncol. Educ. Book.

[149] Pujade-Lauraine E., Ledermann J.A., Selle F., Gebski V., Penson R.T., Oza A.M., Korach J., Huzarski T., Poveda A., Pignata S. (2017). Olaparib tablets as maintenance therapy in patients with platinum-sensitive, relapsed ovarian cancer and a BRCA1/2 mutation (SOLO2/ENGOT-Ov21): A double-blind, randomised, placebo-controlled, phase 3 trial. Lancet Oncol..

[150] Lee J.M., Ledermann J.A., Kohn E.C. (2014). PARP Inhibitors for BRCA1/2 mutation-associated and BRCA-like malignancies. Ann. Oncol..

[151] Zhang L.J., Huang R., Shen Y.W., Liu J., Wu Y., Jin J.M., Zhang H., Sun Y., Chen H.Z., Luan X. (2021). Enhanced anti-tumor efficacy by inhibiting HIF-1alpha to reprogram TAMs via core-satellite upconverting nanoparticles with curcumin mediated photodynamic therapy. Biomater. Sci..

[152] Ning F., Cole C.B., Annunziata C.M. (2020). Driving Immune Responses in the Ovarian Tumor Microenvironment. Front. Oncol..

[153] Ding L., Kim H.-J., Wang Q., Kearns M., Jiang T., Ohlson C.E., Li B.B., Xie S., Liu J.F., Stover E.H. (2018). PARP Inhibition Elicits STING-Dependent Antitumor Immunity in Brca1-Deficient Ovarian Cancer. Cell Rep..

[154] Martincuks A., Song J., Kohut A., Zhang C., Li Y.J., Zhao Q., Mak E., Rodriguez-Rodriguez L., Yu H., Cristea M. (2021). PARP Inhibition Activates STAT3 in Both Tumor and Immune Cells Underlying Therapy Resistance and Immunosuppression In Ovarian Cancer. Front. Oncol..

[155] Wu J.-B., Tang Y.-L., Liang X.-H. (2018). Targeting VEGF pathway to normalize the vasculature: An emerging insight in cancer therapy. OncoTargets Ther..

[156] Seebacher N.A., Stacy A.E., Porter G.M., Merlot A.M. (2019). Clinical development of targeted and immune based an-ti-cancer therapies. J. Exp. Clin. Cancer Res..

[157] Marchetti C., Muzii L., Romito A., Benedetti Panici P. (2019). First-line treatment of women with advanced ovarian cancer: Focus on bevacizumab. OncoTargets Ther..

[158] Oza A.M., Cook A.D., Pfisterer J., Embleton A., Ledermann J.A., Pujade-Lauraine E., Kristensen G., Carey M.S., Beale P., Cervantes A. (2015). Standard chemotherapy with or without bevacizumab for women with newly diagnosed ovarian cancer (ICON7): Overall survival results of a phase 3 randomised trial. Lancet Oncol..

[159] Niu G., Chen X. (2010). Vascular Endothelial Growth Factor as an Anti-Angiogenic Target for Cancer Therapy. Curr. Drug Targets.

[160] Mpekris F., Voutouri C., Baish J.W., Duda D.G., Munn L.L., Stylianopoulos T., Jain R.K. (2020). Combining microen-vironment normalization strategies to improve cancer immunotherapy. Proc. Natl. Acad. Sci. USA.

[161] Wei Y., Song S., Duan N., Wang F., Wang Y., Yang Y., Peng C., Li J., Nie D., Zhang X. (2020). MT1-MMP-Activated Liposomes to Improve Tumor Blood Perfusion and Drug Delivery for Enhanced Pancreatic Cancer Therapy. Adv. Sci..

[162] Heil F., Babitzki G., Julien-Laferriere A., Ooi C.-H., Hidalgo M., Massard C., Martinez-Garcia M., Le Tourneau C., Kockx M., Gerber P. (2021). Vanucizumab mode of action: Serial biomarkers in plasma, tumor, and skin-wound-healing biopsies. Transl. Oncol..

[163] Hidalgo M., Martinez-Garcia M., Le Tourneau C., Massard C., Garralda E., Boni V., Taus A., Albanell J., Sablin M.P., Alt M. (2018). First-in-Human Phase I Study of Single-agent Vanucizumab, A First-in-Class Bispecific An-ti-Angiopoietin-2/Anti-VEGF-A Antibody, in Adult Patients with Advanced Solid Tumors. Clin. Cancer Res..

[164] Russell S., Duquette M., Liu J., Drapkin R., Lawler J., Petrik J. (2015). Combined therapy with thrombospondin-1 type I repeats (3TSR) and chemotherapy induces regression and significantly improves survival in a preclinical model of advanced stage epithelial ovarian cancer. FASEB J..

[165] Scharovsky O.G., Mainetti L.E., Rozados V.R. (2009). Metronomic Chemotherapy: Changing the Paradigm That More Is Better. Curr. Oncol..

[166] Fotopoulou C. (2014). Limitations to the use of carboplatin-based therapy in advanced ovarian cancer. Eur. J. Cancer Suppl..

[167] Aston W.J., Hope D.E., Nowak A.K., Robinson B.W., Lake R.A., Lesterhuis W.J. (2017). A systematic investigation of the maximum tolerated dose of cytotoxic chemotherapy with and without supportive care in mice. BMC Cancer.

[168] Matuszewska K., Ten Kortenaar S., Pereira M., Santry L.A., Petrik D., Lo K.M., Bridle B.W., Wootton S.K., Lawler J., Petrik J. (2022). Addition of an Fc-IgG induces receptor clustering and increases the in vitro efficacy and in vivo anti-tumor properties of the thrombospondin-1 type I repeats (3TSR) in a mouse model of advanced stage ovarian cancer. Gynecol. Oncol..

